# Nutritional timing and stress biology: intermittent fasting as a hormetic signal for adaptation

**DOI:** 10.3389/fnut.2026.1778896

**Published:** 2026-05-07

**Authors:** Olha Strilbytska, Oleh Lushchak

**Affiliations:** 1 Biomedical Research Center, Carpathian National University, Ivano-Frankivsk, Ukraine; 2 Department of Biochemistry and Biotechnology, Vasyl Stefanyk Carpathian National University, Ivano-Frankivsk, Ukraine; 3 Kyiv School of Economics, Kyiv, Ukraine; 4 Research and Development University, Ivano-Frankivsk, Ukraine

**Keywords:** hypothalamic–pituitary–adrenal (HPA) axis, intermittent fasting, metabolism, nutrition, stress

## Abstract

Intermittent fasting (IF) and time-restricted eating (TRE) are recognized as metabolic interventions that link energy balance regulation with influence on stress-related physiological and neuroendocrine processes. Accumulating evidence suggests that IF acts as a mild, controllable stressor that triggers adaptive cellular and systemic responses. Central mechanisms that are involved in these effects are nutrient-sensing pathways, including AMP-activated protein kinase, sirtuins, the target of rapamycin (TOR), and insulin signaling pathways, which collectively coordinate metabolic flexibility and stress adaptation. IF has been shown to modulate hypothalamic–pituitary–adrenal (HPA) axis activity and promote redox and inflammatory homeostasis. This review discusses both preclinical and clinical studies examining the effects of IF and TRE on stress biology and mental health, which frequently report heterogeneous and, in some cases, contradicting effects. Reported effects may vary depending on study design, experimental model, phenotype, and fasting protocol. By integrating data from experimental research, this review highlights the bidirectional interaction between nutritional timing and stress biology and emphasizes that hormesis is a potential mechanism underlying stress resilience. This review also emphasizes the need to carefully balance potential benefits against risks when considering IF interventions for stress resilience and mental health maintenance.

## 1 Introduction

Biologically, stress is defined as a state in which homeostasis of the body is disrupted by internal or external factors (1). Stress is associated with the activation of the hypothalamic–pituitary–adrenal (HPA) axis (general adaptation syndrome—defined by Selye), but the modern interpretation is much broader and includes reactions even in cells and organisms with simpler structures (2). There are several types of stress based on its intensity: low stress or insufficiently challenging stress (sustress), optimal stress (eustress), and excessive stress (distress) (2). Moderate stress (eustress) can stimulate the body, promoting adaptation and strengthening defense mechanisms, while excess of stress can suppress physiological functions. In particular, an optimal level of stress activates hormesis, which is the mechanism of a biological response to a low dose of a stressful factor that stimulates protective mechanisms, while a high dose may have a harmful effect (3, 99). Therefore, the stress response of the body can be both “beneficial,” providing increased resistance to further challenges, and “harmful” when the burden is excessive. Stress exposure is closely related to several psychiatric disorders, including major depressive disorder, bipolar disorder, various types of anxiety disorders, and posttraumatic stress disorder (PTSD) (4). Understanding molecular mechanisms by which stress response influences risk for mental health problems and potential interventions to decrease and prevent chronic stress disorders would contribute to more precise and effective strategies for mental health prevention and intervention.

Intermittent fasting (IF) and time-restricted eating (TRE) are dietary patterns that have been shown to induce metabolic stress. These fasting paradigms differ in duration, timing, and might have different physiological impacts. IF involves cycling between periods of fasting and eating, while TRE restricts food intake to a daily time window (8–10 h per day) (5). Alternate-day fasting (ADF) alternates fasting days, with minimal caloric intake, and ad libitum feeding days. Ramadan fasting involves daily restriction in food and drink from morning to sunset for approximately a month, which causes circadian and nutritional challenges distinct from TRE or ADF (6). These regimens significantly reduce the period of nutrient intake and force the body to switch to alternative energy sources. In particular, under IF conditions, the metabolism switches from glucose metabolism to fat burning and the generation of ketone bodies (7). This process is conceptualized as Mattson Cyclic Metabolic Switching and reflects the organism’s adaptive transition between fed (anabolic) and fasting (catabolic) metabolic states. This metabolic switch activates adaptive cellular responses through the activation of autophagy, adenosine monophosphate-activated protein kinase (AMPK) signaling pathways, and alteration of the expression of certain genes. It was previously shown in the study by Mattson (8) that the effects of IF are caused by cyclic metabolic switching, which leads to the consecutive activation of cellular defense pathways during fasting and growth and recovery pathways during eating. Therefore, IF and TRE act as controlled metabolic stressors that generally increase metabolic plasticity and stress resistance (8).

The process of hormesis may be a potential explanation for the beneficial effects of IF and TRF. Hormesis is an adaptive response to a moderate dose of a stressor that can stimulate protective mechanisms, whereas a large dose suppresses it (99). In this context, IF and TRE are considered examples of hormetic stress. Indeed, IF and TRE regimens involve cyclic periods of fasting that “train” metabolic pathways, forcing them to restore energy. Experimental studies confirm this hypothesis and show that calorie restriction (CR) and IF in mice induce a moderate adaptive cellular stress response and increase resilience (9). It was shown that IF activates signaling pathways AMPK via increased AMP/ATP ratios during fasting (10). Moreover, the IF-induced activation of AMPK and inhibition of target of rapamycin (TOR) trigger autophagy (11). Low-energy state under IF can trigger silent information regulator-1 (SIRT1) that plays an important role in the growth and differentiation of neurons (12). The nuclear factor erythroid 2-related factor 2 (Nrf2) was shown to be involved in energy restriction responses to starvation (13). Nrf2 influences multiple biochemical pathways, including cellular antioxidant responses and the control of inflammatory processes (14). Using *Caenorhabditis elegans*, it was shown that a single period of fasting enhances Heat Shock Factor 1 (HSF-1) activity, enhances stress resistance and extends lifespan (15). Despite significant progress in the study of IF, it is still unclear which molecular factors make IF-induced metabolic stress beneficial. In addition, it remains unclear how signaling pathways are activated during IF (specifically AMPK, mTOR, Nrf2) and interact with the mechanisms underlying stress-induced pathological changes. Therefore, the current review aimed to summarize the latest knowledge about the relationship between IF and stress response mechanisms. We discussed how IF acts as a metabolic stressor, which adaptive signaling pathways are involved, and how these changes jointly increase the resilience of the body. Based on the latest preclinical and clinical data, we focused on the potential for using this knowledge in clinical and practical recommendations.

## 2 Methodology

A structured literature search was performed in PubMed, Google Scholar, Scopus, and Web of Science, covering the period from 2000 to 2026. Search terms included combinations of “intermittent fasting,” “time-restricted eating,” “alternate-day fasting,” “Ramadan fasting,” “metabolic stress,” “hormesis,” and “cellular adaptation.” Additional references were identified through manual screening of the bibliographies of selected papers. Only studies published in English were considered. Exclusion criteria included non-peer-reviewed reports, conference abstracts, dissertations, and duplicate publications. The extracted information included study design, population or experimental model, intervention type and duration, and primary outcomes related to metabolic and stress-biology mechanisms.

## 3 The biology of stress and resilience

Various brain regions and pathways are involved in stress response and resilience. The amygdala is an almond-shaped brain area that plays a primary role in stress response, acting as an emotional sensor of stress (16). The initial stage of the stress response is formed through the complex interaction of individual amygdala nuclei, in particular the lateral, basal, and central complexes. Every other amygdala subnucleus receives signals from sensory systems and limbic structures. For example, the basolateral nucleus integrates cortical and hippocampal information to assess the salience of potential threats, while the central nucleus transmits output signals to the hypothalamus to initiate autonomic and endocrine components of the stress response (16). The autonomic component of stress response involves activation of the sympathetic nervous system (SNS) with inhibition of the parasympathetic nervous system (PSNS). Stress-induced activation of SNS triggers increased secretion of norepinephrine (noradrenalin) and epinephrine (adrenalin) from the adrenal glands (17). Released epinephrine and norepinephrine triggers reaction “fight or flight” by binding to *α*- and *β*-adrenergic G-protein-coupled receptors in the central nervous system (CNS) and peripheral organs and activation intracellular cAMP signaling that rapidly triggers smooth and cardiac muscle contraction, vasoconstriction, increased heart rate, blood pressure, and breathing, enhanced skeletal muscle blood flow, sodium retention, glycogenolysis and gluconeogenesis, lipolysis, elevated oxygen consumption, and thermogenesis (17).

Simultaneously with the SNS activation, the hypothalamus initiates a slower but longer-lasting hormonal mechanism such as HPA axis (18). Paraventricular nucleus of the hypothalamus releases into the circulation corticotropin-releasing hormone (CRH) that triggers the secretion of adrenocorticotropic hormone (ACTH) by the pituitary gland. ACTH triggers the release of glucocorticoid (GC) stress hormones (cortisol in humans and corticosterone in rodents) from the adrenal cortex into the circulation (19). Cortisol regulates its own production through negative feedback and by inhibiting the sensitivity of glucocorticoid receptors (20).

The medial prefrontal cortex (mPFC) is another brain structure that plays an important role in the modulation of autonomic and neuroendocrine stress regulatory systems. The mPFC receives a lot of different signals from the amygdala, hippocampus, and hypothalamus, among others, and, in turn, integrates and regulates the behavioral state of the organism. PFC has an inhibitory effect on threat responses and fear extinction, partly by regulating autonomic output and enhancing parasympathetic tone (21).

Stress-induced elevated secretion of GC results in an increase in metabolic activity that is associated with the generation of reactive oxygen species (ROS) (22–24). Elevated ROS generation and concomitant decrease in capacity of the antioxidant systems of the cell to neutralize ROS lead to oxidative stress (25). ROS disrupt cellular function, damage DNA, lipids, and proteins within the cell. Neurons and glia rely on their antioxidant systems to maintain redox balance (26). Enzymatic antioxidants, including superoxide dismutases (SODs), catalase (CAT), and glutathione peroxidases (GPx), together with non-enzymatic antioxidants such as glutathione (GSH), neutralize ROS and prevent oxidative damage (27). Activation of transcription factors such as Nrf2 further enhances the expression of antioxidant and cytoprotective genes, enabling neurons to resist oxidative challenges induced by HPA axis activation (28). Importantly, ROS are not only mediators of cellular damage but also act as signaling molecules that regulate adaptive stress responses. In this context, IF may act as a metabolic intervention that modulate redox homeostasis.

## 4 Intermittent fasting as a controlled metabolic stressor

Intermittent fasting is considered to be an alternative dietary approach to calorie restriction (CR) and has gained significant attention due to its ability to influence fundamental biochemical pathways regulating cellular metabolism. The most studied IF regimens are time-restricted eating (TRE) and alternate-day fasting (ADF). TRE involves consuming food within a specific time window of the day without significantly reducing overall calorie intake, which allows metabolic processes to be synchronized with the internal circadian clock (29). ADF offers an alternation of feeding and fasting days. All of these modes were shown to simulate a state in cells that triggers specialized adaptation programs aimed at maintaining energy homeostasis and increasing stress resistance (30). IF plays an important role in the modulation of metabolism, and reduction of inflammation and oxidative stress while activating adaptive cellular mechanisms, including autophagy and anti-stress pathways (31). These processes may be involved in maintaining the functional integrity of the brain and slowing down age-related cognitive impairment. In addition, IF causes a positive effect on the cardiovascular system. Indeed, cycling between periods of fasting and eating within IF regime leads to normalization of blood pressure and reduction of cholesterol and triglyceride levels, which together reduce the risk of developing cardiovascular disease (32, 33). The positive effects of IF are driven by its influence on metabolism. By triggering metabolic switching, IF facilitates weight loss, minimizes visceral adiposity, cardiometabolic risk, and glucose homeostasis (30).

Physiological effects of intermittent fasting can be considered as a sequence of interdependent phases. The response phase is characterized by acute catabolic processes and activation of stress-response pathways, which is consistent with the metabolic switch (8). This phase is followed by an adaptation phase that involves strategic allocation of cellular resources and metabolic reprogramming (100). Finally, the recovery phase is marked by anabolic processes during the refeeding period (34). However, recovery is not a passive, time-dependent process because it requires bioenergetic support and availability of metabolic substrates to provide anabolic repair and functional restoration. Mitochondria play a central role in this phase by determining cellular ATP generation and biosynthetic capacity (35). The coordinated interplay between these phases underlies the hormetic benefits of IF and supports improved metabolic flexibility and cellular resilience.

**Figure 1:**
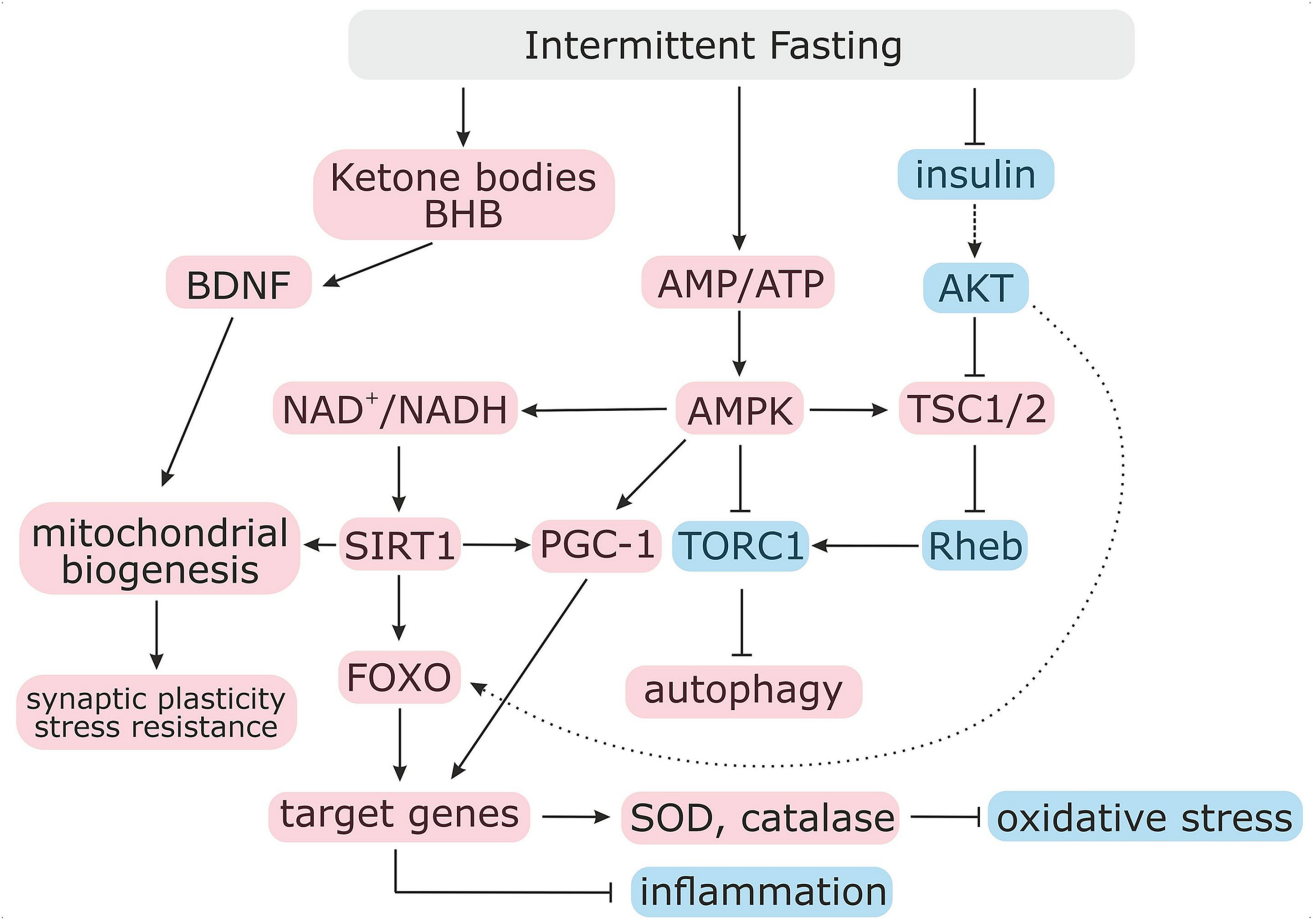
Effects of intermittent fasting on metabolic and stress-response signaling pathways. Intermittent fasting induces a metabolic shift characterized by reduced nutrient and growth factor availability, leading to suppression of insulin/IGF-1 signaling and downstream inhibition of mTOR activity. Decreased mTOR signaling relieves inhibition of autophagy, thereby promoting cellular recycling. In parallel, energy deficit activates AMPK, which further inhibits mTOR and stimulates autophagy through phosphorylation of key regulatory proteins. AMPK activation also enhances NAD⁺ availability, facilitating SIRT1 activation. SIRT1, together with AMPK, promotes deacetylation and activation of PGC-1*α*, resulting in increased mitochondrial biogenesis and oxidative metabolism. In neural tissues, these metabolic adaptations are associated with increased expression of brain-derived neurotrophic factor BDNF, linking intermittent fasting to improved neuronal resilience and synaptic plasticity. BDNF, brain-derived neurotrophic factor; IGF-1, insulin growth factor-1; TOR, target of rapamycin; AMPK, AMP-activated protein kinase; SIRT1, Sirtuin 1; PGC-1α, peroxisome proliferator-activated receptor-*γ* (gamma) coactivator 1-α (alpha).

One of the key mechanisms that is involved in the beneficial effects of IF is the hormetic effect, which implies the activation of protective cellular systems in response to moderate and controlled stress. Energy stress during IF increases the AMP/ATP ratio and activates a number of signaling pathways, including AMPK, which acts as a key cellular energy sensor. Activation of AMPK leads to inhibition of anabolic processes, including lipid, protein and glycogen synthesis, with concomitant activation of catabolic processes that are associated with energy production (in the form of ATP) (36). Moreover, activated AMPK promotes autophagy through ULK1 phosphorylation at Ser317 and Ser777 (37). In contrast, activation of target of rapamycin (TOR) signaling by nutrients and amino acids leads to ULK1 phosphorylation in Ser757, which prevents ULK1 and AMPK interaction and, in turn, autophagy inhibition (37). Nutrient deprivation leads to TOR signaling inhibition that results in autophagy activation (38). AMPK activation by energy deficiency triggers mitochondrial biogenesis via two mechanisms. The first mechanism involves AMPK-dependent phosphorylation and enhancing the transcriptional activity of Peroxisome Proliferator-Activated Receptor Gamma Coactivator-1 Alpha (PGC-1α) (39). In parallel, increasing NAD⁺ levels occurs and stimulates SIRT1-dependent deacetylation of PGC-1α. These coordinated mechanisms trigger the expression of genes that play a role in ribosome biogenesis (Figure 1).

Cyclical changes in energy availability increase mitophagy and renewal of the mitochondrial population, which overall reduces oxidative stress and increases resistance to metabolic overload (40). Such adaptations increase cellular resilience to both metabolic and psychological stress.

## 5 Mechanistic links between fasting and stress biology

Intermittent food intake is considered a metabolic stressor that activates the hypothalamic–pituitary–adrenal (HPA) axis. Studies have shown that even a single day of fasting activates the HPA axis, which leads to increased cortisol levels in blood plasma (41, 42). For example, an 8-day water fast significantly increases circulating cortisol levels (43). Simultaneously, a significant decrease in leptin is observed, which also activates the HPA axis (43). The decrease in blood glucose levels during fasting also causes additional activation of the HPA axis, as low glycemic levels stimulate the release of cortisol (44, 45). Therefore, IF stimulates cortisol secretion and metabolic shift from glucose to lipids.

Intermittent fasting causes alterations in the autonomic nervous system. In animal models, alternating between fasting and feeding leads to a sustained decrease in heart rate and blood pressure (46). Similar beneficial effects IF on cardiovascular autonomic regulation were observed in humans. Indeed, an 8-week TRF 16:8 regimen (16 h of fasting) increases the standard deviation of normal-to-normal (NN) intervals (SDNN) and root mean square of successive differences (RMSSD), heart rate variability (HRV) parameters, with concomitant decrease in resting heart rate in healthy adults (47). These effects could be associated with IF-induced increased levels of brain-derived neurotrophic factor (BDNF), which improves the cholinergic activity of cardiovagal neurons in the brainstem (48). Decreased level of BDNF in the peripheral nervous system was shown to result in cognitive performance decline (49). Therefore, IF shifts the vegetative balance toward parasympathetic dominance, which is reflected in better adaptation of the cardiovascular system to stress.

Fasting leads to a metabolic “switch.” Reduced glucose availability leads to enhanced lipid catabolism and increased ketogenesis, resulting in the production of ketone bodies, including *β*-hydroxybutyrate (BHB) (50). BHB serves as an effective energy substrate and signaling molecule (51). BHB increases mitochondrial biogenesis through the activation of PGC-1*α* (52). Moreover, BHB inhibits histone acetyltransferases and stimulates the expression of the neurotrophic factor BDNF (53) that stimulates synaptic plasticity and neuron survival. As a modulator of antioxidant defense, BHB activates the Nrf2 transcription factor, which increases the synthesis of key antioxidant enzymes, including superoxide dismutase (SOD2), catalase, etc. (54). This effect is also linked to the activation of AMPK, which suppresses mTOR during energy deficiency and switches cells into a state of utilizing their own reserves (55). In general, metabolic changes during IF contribute to improved mitochondrial function and reduced oxidative stress.

Intermittent fasting also exerts an anti-inflammatory effect (56). It suppresses the expression of pro-inflammatory cytokines, including interleukin 6 (IL-6) and tumor necrosis factor α (TNF-α) (56, 57). This indicates a reduction in systemic inflammation during fasting. IF also results in a decrease in markers of oxidative stress. It was shown that 10 days of fasting reduced the concentration of lipid peroxidation markers (TBARS/MDA) and increased the total antioxidant capacity of blood plasma of volunteers (58). In addition, the adrenal hormone cortisol was shown to enhance lipolysis, reducing excess fat deposits (59). It is important to note that ketone bodies (especially BHB) directly inhibit the activity of NLRP3 inflammasome (60), which acts as a key sensor of intracellular stress. Inhibition of NLRP3 inflammasome causes reduction of the synthesis of pro-inflammatory cytokines IL-1β and IL-18 (61).

The induction of molecular factors that enhance the antioxidant defence of the body is also observed during IF. It has been shown that prolonged fasting stimulates the expression of superoxide dismutases 1 and 2, and catalase in brain cells of gerbils (62). According to some studies, 8 weeks of 16:8 IF diet increases the level of catalase in the blood serum of postmenopausal women with rheumatoid arthritis (63). The increase in these enzymes correlates with a decrease in lipid peroxidation and enhanced glutathione regeneration, which generally improves redox homeostasis. The main regulators of redox homeostasis are Nrf2 signaling as well as AMPK-mTOR-SIRT1 signaling axis. Energy deficiency during IF activates AMPK and SIRT1 (64), suppressing mTOR, which promotes autophagy (65). IF has been demonstrated to be a powerful activator of autophagy as it increases the activity of genes and proteins associated with the apoptotic and lysosomal pathways, including upregulation of PINK1 and Parkin in mitophagy (11). Effective elimination of defective proteins and organelles within autophagy limits oxidative stress at the cellular level.

A growing body of research has investigated the effects of intermittent fasting on stress responses in both humans and animal models. Human studies have primarily focused on physiological and psychological outcomes, while preclinical models provide mechanistic insights into the cellular and molecular pathways involved.

### 5.1 Preclinical evidence from animal models

Rodent models are instrumental in elucidating the effects of IF on HPA axis regulation, neuroplasticity, and oxidative stress. Furthermore, findings from animal studies provide a translational foundation that helps to properly design human trials and facilitate the interpretation of physiological and behavioral outcomes observed in clinical studies. Table 1 provides an overview of preclinical studies investigating the effects of IF on stress responses, highlighting the experimental models used, IF regimen applied, stress paradigms employed, and the main physiological, neuroendocrine, and behavioral outcomes. In the study by Wan et al. (33), the physiological and biochemical effects of IF in rats were investigated. Five-month-old male rats exposed to fasting every other day for 6 months had significantly lower heart rate (HR) and blood pressure (BP), as well as reduced adrenocorticotropic hormone (ACTH) and corticosterone (33). Exposures to the immobilization and swim stressors lead to increased ACTH, corticosterone and epinephrine levels in ad libitum rats; however, there was no significant increase in the levels of ACTH, corticosterone and epinephrine in rats exposed to IF (33). Similarly, elevated levels of stress hormones, including norepinephrine and serotonin, in rats after the first day of ADF were demonstrated in the study by Abdel-Rahman and colleagues (98). Gamma-amino butyric acid, glycine and glutathione levels were elevated in male Wistar rats exposed to IF (98). It is interesting to note that BDNF levels were higher after 1 day of IF in the hippocampus, which may indicate promoted stress resilience and synaptic plasticity, and decreased after 15 days of IF, suggesting that prolonged IF triggers homeostatic downregulation of neurotrophic signaling (98).

**Table 1:** Overview of preclinical studies on the effects of intermittent fasting (IF) on stress response.

Animal model	IF regime	Stress markers	Effects	Reference
Male Sprague–Dawley rats, five-month-old	Every other day fasting for 6 months	ACTH, corticosterone, HR, BP	Lower HR/BP in the IF group; ↑ACTH and corticosterone levels; but stress-induced increases in epinephrine were reduced	(33)
Male Wistar rats, 2-3-month-old	ADF for 15 days	Brain neurotransmitters (dopamine, norepinephrine, serotonin, GABA, glycine, etc.); oxidative and antioxidant markers in the brain (MDA, NO, GSH); BDNF.	↓ body weight; after 1 day of IF, decreased brain oxidative stress (↓MDA, NO) and increased antioxidants (↑GSH); altered neurotransmitters (↑NE, 5-HT, GABA, ↓glutamate); BDNF increased after 1 day but decreased after 15 days of IF.	(98)
Male C57BL/6 N mice	ADF for 9 days	Behavioral tests (locomotion, feeding rate), inflammatory cytokines (TNF-α, IL-6, MCP-1), plasma corticosterone	IF enhances the immune response to the viral mimetic Poly(I: C), leading to increased cytokine levels and elevated “sickness” behavior; ↑ hypothalamic NPY ↑ plasma corticosterone levels	(66)
Male and female Wistar rats aged 24 days	Alternate-day fasting 8 weeks	Behavioral tests OF, object recognition, and EPM	IF led to anxiety-like behavior ↓ entries and reduced time in the center of the OF and open arms of the EPM) and impaired memory performance (↓ object recognition index); regular physical exercise mitigated these adverse effects.	(67)
Adult Wistar rats	TRF; ADF; ADMF for 3 weeks	Behavioral tests - OF, EPM, Splash-test	ADF and ADMF groups had the lowest weight over the weeks and ↓ depressive-like behavior, while TRF ↓ anxiety-like behavior	(69)
Male C57BL/6 mice	ADF for 16 or 5 weeks	Behavioral tests - OF, EPM; expression of *Sirt1*, MAO-A in the hippocampus, the expression of 5-HT	↓ body weight, more BAT, ↓ anxiety behavior, ↓ pathological damage in the hippocampus, ↓expression of *Sirt1* ↓ MAO-A protein and ↑ 5-HT levels in the hippocampus	(68)

ACTH, Adrenocorticotropic hormone; HR, heart rate; BP, blood pressure; ADF, alternate-day fasting; GABA, gamma-aminobutyric acid; MDA, malondialdehyde; GSH, glutathione; BDNF, brain-derived neurotrophic factor; TNF-α, tumor necrosis factor-alpha; IL-6, interleukin-6, MCP-1, monocyte chemotactic protein-1; NPY, neuropeptide Y; OF, open field; EPM, elevated plus maze; TRF, time-restricted feeding; ADMF, alternate-day modified fasting; MAO-A, monoamine oxidase A; 5-HT, 5-hydroxytryptamine; BAT, brown adipose tissue.

Behavioral manifestations of IF in animal models depend on phenotype. It was shown that TLR3 stimulation by the injection of viral mimic Poly(I: C) leads to a decrease in locomotor activity and exploratory rearing behavior in the open field (OF) test, as well as a decrease in water and food intake and 9 days of alternate day fasting exacerbate these effects (66). Elevated cytokine expression manifested by enhanced secretion of IL-6 and IFN-*α* was found in the Poly(I: C)-treated IF group, which may be one of the potential mechanisms involved in sickness behavior in mice after IF (66). Moreover, elevated corticosterone level and expression of neuropeptide Y (NPY) mRNA was shown in the hypothalamus of IF mice (66). The study by Braz et al. (67) demonstrated that 8 weeks of alternate-day fasting causes anxiety-like behavior and symptoms of cortical spreading depression (CSD) in rats, which is manifested by decreased time spent on the central part of the OF arena and open arms of the elevated plus maze (EPM). Impaired memory was observed in the novel object recognition test in rats that were exposed to IF (67). However, other studies showed beneficial effects of IF in alleviating stress symptoms in rodents (68, 69). Different dietary regimens were investigated, including caloric restriction (CR), TRF, ADF, and alternate-day modified fasting (ADMF) in their effects on behavior and brain tissues (69). Using a set of behavioral tests such as OF, EPM, Splash-test, it was demonstrated the decreased anxiety- and depression-like behavior in rats exposed to ADMF and ADF for 4 weeks (69). Mice exposed to ADF for 16 weeks showed reduced body weight and less anxiety behavior in the OF and EPM tests (68). There are some controversial studies about damage in the hippocampus caused by IF, as a study by Soares et al. (69) showed some damage in the brain tissues of rats, such as the central cortex and hippocampus, after TRF, ADF, and ADMF. However, the study by Hu et al. (68) demonstrated less damage in the hippocampus, as well as lower expression of Sirt1 and Monoamine oxidase A protein in the hippocampus in obese mice.

### 5.2 Clinical evidence in humans

Several clinical studies reported beneficial effects IF on mental health and sleep quality (70–72) (Table 2). Significant improvement in fatigue, mood, and sleep was found at mid Ramadan, the last days of Ramadan, and 1 week after Ramadan in healthy males (70) as well as in participants of both sexes (71). BDNF and cortisol levels significantly decreased in healthy participants on the last days and 1 week after Ramadan fasting (72). Even a short-term (24-h) fasting intervention significantly modulates the diurnal secretion patterns of cortisol and dehydroepiandrosterone (DHEA) in the saliva of individuals with obesity (73). Five-day fasting leads to decreased cortisol levels in the urine of healthy men (74). However, in healthy volunteers, 72 h of fasting significantly increased cortisol and norepinephrine in the blood and was accompanied by an increase in somatic depressive symptoms assessed by Beck’s Depression Inventory (BDI)-2 sum-score (45).

**Table 2:** Clinical studies on intermittent fasting (IF) examining HPA axis activity, oxidative stress markers, and psychological assessments.

Participants	Experimental design	IF regime	Parameters HPA/OS/psychological tests	Effects	Reference
Patients with MDD (19–59 years) and healthy individuals (22–66 years)	Cross-Sectional Study	72 h fasting	HPA hormones: norepinephrine, aldosterone, cortisol; psychological testing (BDI-II: total score and subscales).	In MDD patients, 72-h IF reduced cognitive-affective symptoms but increased somatic symptoms; BDI-II scores decreased in those with severe depression. Symptom changes correlated with BDNF alterations	(45)
Healthy men and women (most of them are students of HMS), 24–26 years	Observational study	Ramadan fasting, 17–18 h per day during 30 days	Cortisol, BNDF, mood, IGF-1, IL-8, MMP-9, and myoglobin	↓ Cortisol and BDNF levels on days 15–30 compared to pre-fasting values. Improved mood and well-being	(72)
Healthy men, (most of them are students of HMS), 24–26 years	Prospective controlled study	Ramadan fasting, 17–18 h per day during 30 days	Mood, fatigue, and health-related Quality of Life	improvement in fatigue, mood, and sleepiness ↓ body weight, ↓ BMI ↓ muscle mass ↓ fat mass	(70)
Healthy men and women (most of them are students of HMS), 24–26 years	Prospective controlled study	Ramadan fasting, 17–18 h per day during 30 days	Mood, fatigue, and sleep pattern	improvement in fatigue and mood	(71)
Obese patients, 26–70 years	Randomized Controlled Trial	25–30% reduction in the daily caloric supply	cortisol and DHEA levels	IF increased the amplitude and shifted the phase of the diurnal cortisol rhythm, ↑DHEA levels.	(73)
Healthy men and women, 34.0 ± 11.7 years	Pilot Study	16/8 TRE for 4 weeks	Questionnaire on sleep (PittsburghSleep Quality Index) and stress (Perceived Stress Scale)	↓stress levels. No significant changes in sleep quality	(75)
Adults With Overweight or Obesity, 30–60 years	Randomized Clinical Trial	16/8 TRE for 12 weeks	Mood dimensions-depression, anxiety, and stress-and quality of life	No changes in sleep, mood, or quality of life	(76)
Healthy men, 30–70 years	Randomized Clinical Trial	8 days of water-only fasting	cortisol, TAS, and lipid peroxidation	↑cortisol, improved TAS, and ↓lipid peroxidation	(43)
Twenty-six healthy adults, 25–30 years	Randomized Clinical Trial	16/8 TRE for 50 days	BMI, metabolic parameters, SAS, SDS, and fMRI connectivity of the amygdala	↓BMI, ↓glucose and ↓insulin concentrations, ↓insulin resistance, and ↓anxiety scores	(77)

MDD, major depressive disorder; HPA axis, hypothalamic–pituitary–adrenal axis; BDI-II, Beck Depression Inventory-Second Edition; BDNF, brain-derived neurotrophic factor; IGF-1, insulin growth factor-1; IL-8, interleukin 8; MMP-9, matrix metalloproteinase; HMS, Harvard Medical School; BMI, body mass index; DHEA, dehydroepiandrosterone; TRF, time-restricted feeding; TAS, total antioxidant status; SAS, Self-Rating Anxiety Scale; SDS, Self-Rating Depression Scale; fMRI, resting-state functional magnetic resonance imaging.

A pilot study by Bains et al. (75) investigated the effects of 4 weeks of TRF on body composition, stress levels, sleep quality, hunger levels, and quality of life. Using the Perceived Stress Scale by Cohen (PSS-10) scale, it was demonstrated a significant reduction in stress and improvement of overall health in healthy individuals after 4 weeks of TRF (75). However, there was no significant change in sleep quality according to the Pittsburgh Sleep Quality Index (PSQI) after TRF (75). No significant changes in sleep, mood, or quality of life were found in adults with overweight or obesity after 2 weeks of 16/8 TRF (76).

The mechanisms by which IF can enhance human health, stress resistance and overall well-being are associated with changes in metabolic parameters, pro-inflammatory markers, and oxidative stress (43). After 8-days of water-only fasting, it was observed improved total antioxidant status (TAS), and reduced lipid peroxidation, however, cortisol levels were elevated in middle-aged men (43). Anxiety scores within the Self-Rating Anxiety Scale (SAS) decreased significantly after 16/8 TRF for 50 days in healthy adults (77). However, there were no significant changes in depression scores within the Self-Rating Depression Scale (SDS) after TRF (77). After 30 and 50 days of TRF, a marked attenuation of functional connectivity was observed between the postcentral gyrus of the primary somatosensory cortex and the laterobasal subdivision of the amygdala, a neural network involved in affective regulation, stress responsiveness, and feeding-related reward processes (77).

The reviewed molecular, preclinical, and clinical evidence indicates that IF can modulate neural and hormonal pathways relevant to mood, stress, and cognition, yet the outcomes are highly variable across studies. This variability emphasizes the need for careful consideration of individual differences and risk–benefit balance in applying IF for mental health. Moreover, psychological outcomes observed during fasting interventions could be influenced by multiple interacting factors. For example, Ramadan fasting not only involves nutrient restriction but also is associated with circadian changes that may affect mood and perceived stress. In addition, alterations in sleep patterns and weight loss can contribute to observed changes in psychological states.

## 6 Physiological and psychological risks of fasting

IF triggers a cascade of neuroendocrine and metabolic responses, including activation of the HPA axis, the sympathoadrenal system, and AMPK-mTOR-SIRT1 signaling pathways, which can be both adaptive and potentially disadaptive. The balance between a beneficial hormonal response and excessive stress is determined by the individual biological traits of the organism, which causes significant interindividual variability in the effects of fasting. The response to IF differs significantly between males and females (78–81), and also changes with age (82). Fasting can be considered as a strong stressor by the body of females of reproductive age, leading to dysregulation of the reproductive system manifested by decreased luteinizing hormone, estrogen levels (83). The activation of the HPA axis in response to fasting is more pronounced in older individuals. It was demonstrated that older volunteers experience a greater increase in cortisol than younger volunteers during several days of fasting (84).

An important but often underestimated factor is chronotype and circadian organization of metabolism. Persons with an “evening” chronotype statistically have a higher risk of developing metabolic disorders such as type 2 diabetes mellitus (85). Late food intake and early fasting in individuals with “evening” chronotype can lead to desynchronization of the circadian clock, potentially enhancing stress response (32, 86). Conversely, individuals with a “morning” chronotype adapt more easily to a morning fasting regimen (87).

Metabolic status is another important determinant of the response to IF. Persons with obesity, metabolic syndrome, or insulin resistance often demonstrate positive metabolic changes during IF (88, 89). Activating AMPK and ketogenesis during fasting inhibits insulin resistance, promotes survival of *β* cells, and lowers blood sugar (90). Fasting usually reduces body weight and improves glycemic control in patients with metabolic syndrome (91) and patients with type 2 diabetes (92, 93). However, in individuals with low body mass, IF may cause excessive catabolism, decreased muscle mass, and fatigue (94). These effects are particularly evident in the absence of adequate protein intake and are mediated by AMPK activation, reduced insulin/IGF-1 and TOR signaling, and elevated cortisol levels. Moreover, the composition of the gut microbiota can influence the response to IF. During fasting, there is an increase in the population of bacteria such as *Prevotella, Lactobacillus*, and *Anaerostipes* that produce short-chain fatty acids (SCFA) (95). This can have an anti-inflammatory effect and promote metabolic health, positively affecting liver function, glycemia, and immunity (96). However, the initial state of the microbiota can also modulate IF response (97).

The effects of intermittent fasting may be modulated by the presence of severe psychological disorders in individuals. In patients with major depressive disorder (MDD), 72-h fasting caused HPA activation and an increase in somatic symptoms, such as anorexia, appetite disorders, but in patients with severe, resistant depression, a decrease in cognitive-affective symptoms was observed (45). It is worth noting that individuals with current or past eating disorders, such as anorexia or bulimia, are systematically excluded from IF studies (45), because any dietary restrictions may trigger a recurrence of pathological eating behavior. Clinical contraindications for IF include severe mental disorders, including acute psychosis, bipolar disorder, and severe anxiety. IF is also not recommended for pregnant women due to the need for adequate nutrition, as well as for children and adolescents due to the need for growth. Considering ethical issues, the implementation of IF in vulnerable patient groups requires careful monitoring. In this case, it may be recommended to consider alternative interventions that increase ketone body levels, including a ketogenic diet or regular exercise without significant stress for the organism.

## 7 Conclusion and future perspectives

Intermittent fasting (IF) functions as a form of physiological training by acting as a mild, hormetic stimulus, enhancing resilience at both cellular and neural levels. Periodic caloric restriction stimulates BDNF production and improves neuronal stress tolerance to psychophysiological challenges. At the systemic level, IF orchestrates coordinated metabolic and endocrine adaptations to maintain cellular homeostasis. Activation of AMPK, inhibition of mTOR, modulation of hormones such as leptin, adiponectin, ghrelin, and cortisol, alongside the induction of autophagy, collectively support energy balance while reducing oxidative and inflammatory stress. For instance, AMPK activation and mTOR suppression promote autophagic clearance of damaged cellular components. Conceptually, these effects represent hormesis as the principle that mild stress enhances resilience. By lowering IGF-1/insulin signaling, IF activates protective hormetic pathways, including enhanced antioxidant defenses and improved mitochondrial function. Such adaptive responses suggest that controlled fasting or similar mild stressors (such as physical exercise or pharmacological modulators) could be promising to prevent stress disorders and enhance cognitive resilience.

Preclinical and clinical studies report heterogeneous effects of IF on stress response, highlighting variability in the effects of IF on mental health and stress resilience across different models and populations. Further research should focus on identifying individual factors such as age, sex, baseline metabolic status, and genetic background that may influence responsiveness to IF, as well as characterizing potential adverse effects. Longitudinal clinical studies that will aim to integrate neuroimaging, hormonal profiling, and cognitive assessments are needed to clarify the optimal duration and regimen of IF interventions. Collectively, these data will elucidate the potential of IF as a preventive or therapeutic strategy for stress-related disorders, cognitive decline, and age-associated functional deterioration.

## References

[1] Buenrostro-Jáuregui MH Muñóz-Sánchez S Rojas-Hernández J Alonso-Orozco AI Vega-Flores G Tapia-de-Jesús A . A comprehensive overview of stress, resilience, and neuroplasticity mechanisms. Int J Mol Sci. (2025) 26:3028. doi: 10.3390/ijms26073028, 40243691 PMC11988468

[2] Lu S Wei F Li G. The evolution of the concept of stress and the framework of the stress system. Cell Stress. (2021) 5:76–85. doi: 10.15698/cst2021.06.250, 34124582 PMC8166217

[3] Calabrese F Molteni R Racagni G Riva MA. Neuronal plasticity: a link between stress and mood disorders. Psychoneuroendocrinology. (2009) 34:S208–16. doi: 10.1016/j.psyneuen.2009.05.014, 19541429

[4] Davis MT Holmes SE Pietrzak RH Esterlis I. Neurobiology of chronic stress-related psychiatric disorders: evidence from molecular imaging studies. Chronic Stress (Thousand Oaks). (2017) 1:2470547017710916. doi: 10.1177/2470547017710916, 29862379 PMC5976254

[5] Strilbytska O Klishch S Storey KB Koliada A Lushchak O. Intermittent fasting and longevity: from animal models to implication for humans. Ageing Res Rev. (2024) 96:102274. doi: 10.1016/j.arr.2024.102274, 38499159

[6] Mindikoglu AL Opekun AR Gagan SK Devaraj S. Impact of time-restricted feeding and Dawn-to-sunset fasting on circadian rhythm, obesity, metabolic syndrome, and nonalcoholic fatty liver disease. Gastroenterol Res Pract. (2017) 2017:1–13. doi: 10.1155/2017/3932491, 29348746 PMC5733887

[7] Mishra A Sobha D Patel D Suresh PS. Intermittent fasting in health and disease. Arch Physiol Biochem. (2024) 130:755–67. doi: 10.1080/13813455.2023.2268301 37828854

[8] Mattson MP. The cyclic metabolic switching theory of intermittent fasting. Nat Metab. (2025) 7:665–78. doi: 10.1038/s42255-025-01254-5, 40087409

[9] Dias GP Murphy T Stangl D Ahmet S Morisse B Nix A . Intermittent fasting enhances long-term memory consolidation, adult hippocampal neurogenesis, and expression of longevity gene klotho. Mol Psychiatry. (2021) 26:6365–79. doi: 10.1038/s41380-021-01102-4, 34031536 PMC8760057

[10] Zambuzzi WF Ferreira MR Wang Z Peppelenbosch MP. A biochemical view on intermittent fasting's effects on human physiology-not always a beneficial strategy. Biol-Basel. (2025) 14:669. doi: 10.3390/biology14060669, 40563920 PMC12190167

[11] Wolska W Gutowska I Wszołek A Żwierełło W. The role of intermittent fasting in the activation of autophagy processes in the context of Cancer diseases. Int J Mol Sci. (2025) 26:4742. doi: 10.3390/ijms26104742, 40429883 PMC12112746

[12] Gomes BAQ Silva JPB Romeiro CFR Dos Santos SM Rodrigues CA Gonçalves PR . Neuroprotective mechanisms of resveratrol in Alzheimer's disease: role of SIRT1. Oxidative Med Cell Longev. (2018) 2018:8152373. doi: 10.1155/2018/8152373, 30510627 PMC6232815

[13] Tebay LE Robertson H Durant ST Vitale SR Penning TM Dinkova-Kostova AT . Mechanisms of activation of the transcription factor Nrf2 by redox stressors, nutrient cues, and energy status and the pathways through which it attenuates degenerative disease. Free Radic Biol Med. (2015) 88:108–46. doi: 10.1016/j.freeradbiomed.2015.06.021, 26122708 PMC4659505

[14] Saha S Buttari B Panieri E Profumo E Saso L. An overview of Nrf2 signaling pathway and its role in inflammation. Molecules. (2020) 25:5474. doi: 10.3390/molecules25225474, 33238435 PMC7700122

[15] Tataridas-Pallas N Aman Y Williams R Chapman H Cheng KJH Gomez-Paredes C . Mitochondrial clearance and increased HSF-1 activity are coupled to promote longevity in fasted *Caenorhabditis elegans*. iScience. (2024) 27:109834. doi: 10.1016/j.isci.2024.109834, 38784016 PMC11112483

[16] Zhang WH Zhang JY Holmes A Pan BX. Amygdala circuit substrates for stress adaptation and adversity. Biol Psychiatry. (2021) 89:847–56. doi: 10.1016/j.biopsych.2020.12.026, 33691931

[17] Chu B Marwaha K Sanvictores T Awosika AO Ayers D. "Physiology, stress reaction". In: StatPearls. Treasure Island (FL): StatPearls Publishing (2025) 31082164

[18] Godoy LD Rossignoli MT Delfino-Pereira P Garcia-Cairasco N de Lima Umeoka EH. A comprehensive overview on stress neurobiology: basic concepts and clinical implications. Front Behav Neurosci. (2018) 12:127. doi: 10.3389/fnbeh.2018.00127, 30034327 PMC6043787

[19] Levone BR Cryan JF O’Leary OF. Role of adult hippocampal neurogenesis in stress resilience. Neurobiol Stress. (2015) 1:147–55. doi: 10.1016/j.ynstr.2014.11.003, 27589664 PMC4721321

[20] Kakehi R Hori H Yoshida F Itoh M Lin M Niwa M . Hypothalamic-pituitary-adrenal axis and renin-angiotensin-aldosterone system in adulthood PTSD and childhood maltreatment history. Front Psychol. (2023) 13:967779. doi: 10.3389/fpsyt.2022.967779, 36699501 PMC9869036

[21] Alexandra Kredlow M Fenster RJ Laurent ES Ressler KJ Phelps EA. Prefrontal cortex, amygdala, and threat processing: implications for PTSD. Neuropsychopharmacology. (2022) 47:247–59. doi: 10.1038/s41386-021-01155-7, 34545196 PMC8617299

[22] Lushchak O Orru M Strilbytska O Berezovskyi V Cherkas A Storey KB . Metabolic and immune dysfunctions in post-traumatic stress disorder: what can we learn from animal models? EXCLI J. (2023) 22:928–45. doi: 10.17179/excli2023-6391, 38023568 PMC10630527

[23] Lushchak O Strilbytska O Koliada A Storey KB. An orchestrating role of mitochondria in the origin and development of post-traumatic stress disorder. Front Physiol. (2023) 13:1094076. doi: 10.3389/fphys.2022.1094076, 36703926 PMC9871262

[24] Strilbytska O Koliada O Lushchak VI Lushchak O. "Posttraumatic stress disorder and the mitochondria". In: Martin CR Preedy VR Patel VB Rajendram R, editors. Handbook of the Biology and Pathology of Mental Disorders. Cham: Springer (2024)

[25] Lushchak VI Duszenko M Gospodaryov DV Garaschuk O. Oxidative stress and energy metabolism in the brain: midlife as a turning point. Antioxidants (Basel). (2021) 10:1715. doi: 10.3390/antiox10111715, 34829586 PMC8614699

[26] Lee KH Kim UJ Lee BH Cha M. Safeguarding the brain from oxidative damage. Free Radic Biol Med. (2025) 226:143–57. doi: 10.1016/j.freeradbiomed.2024.11.019 39547523

[27] Jomova K Alomar SY Alwasel SH Nepovimova E Kuca K Valko M. Several lines of antioxidant defense against oxidative stress: antioxidant enzymes, nanomaterials with multiple enzyme-mimicking activities, and low-molecular-weight antioxidants. Arch Toxicol. (2024) 98:1323–67. doi: 10.1007/s00204-024-03696-4, 38483584 PMC11303474

[28] Goodfellow MJ Borcar A Proctor JL Greco T Rosenthal RE Fiskum G. Transcriptional activation of antioxidant gene expression by Nrf2 protects against mitochondrial dysfunction and neuronal death associated with acute and chronic neurodegeneration. Exp Neurol. (2020) 328:113247. doi: 10.1016/j.expneurol.2020.113247, 32061629 PMC8627637

[29] Chaix A Rynders CA. Time restricted feeding plus exercise: could two be better than one for metabolic health? J Physiol. (2022) 600:699–700. doi: 10.1113/JP281358, 33533524 PMC8329115

[30] Zang BY He LX Xue L. Intermittent fasting: potential bridge of obesity and diabetes to health? Nutrients. (2022) 14:981. doi: 10.3390/nu14050981, 35267959 PMC8912812

[31] Longo VD Mattson MP. Fasting: molecular mechanisms and clinical applications. Cell Metab. (2014) 19:181–92. doi: 10.1016/j.cmet.2013.12.008, 24440038 PMC3946160

[32] Lotti S Dinu M Colombini B Amedei A Sofi F. Circadian rhythms, gut microbiota, and diet: possible implications for health. Nutr Metab Cardiovasc Dis. (2023) 33:1490–500. doi: 10.1016/j.numecd.2023.05.009 37246076

[33] Wan R Camandola S Mattson MP. Intermittent food deprivation improves cardiovascular and neuroendocrine responses to stress in rats. J Nutr. (2003) 133:1921–9. doi: 10.1093/jn/133.6.1921, 12771340

[34] Calabrese EJ Mattson MP. The catabolic - anabolic cycling hormesis model of health and resilience. Ageing Res Rev. (2024) 102:102588. doi: 10.1016/j.arr.2024.102588, 39551161

[35] Lushchak O Gospodaryov D Strilbytska O Bayliak M. Changing ROS, NAD and AMP: a path to longevity via mitochondrial therapeutics. Adv Protein Chem Struct Biol. (2023) 136:157–96. doi: 10.1016/bs.apcsb.2023.03.005, 37437977

[36] Sharma A Anand SK Singh N Dwivedi UN Kakkar P. AMP-activated protein kinase: an energy sensor and survival mechanism in the reinstatement of metabolic homeostasis. Exp Cell Res. (2023) 428:113614. doi: 10.1016/j.yexcr.2023.113614, 37127064

[37] Kim J Kundu M Viollet B Guan KL. AMPK and mTOR regulate autophagy through direct phosphorylation of Ulk1. Nat Cell Biol. (2011) 13:132–41. doi: 10.1038/ncb2152, 21258367 PMC3987946

[38] Senapati PK Mahapatra KK Singh A Bhutia SK. mTOR inhibitors in targeting autophagy and autophagy-associated signaling for cancer cell death and therapy. Biochim Biophys Acta Rev Cancer. (2025) 1880:189342. doi: 10.1016/j.bbcan.2025.189342, 40339669

[39] DiNicolantonio JJ McCarty MF O'Keefe JH. Nutraceutical activation of Sirt1: a review. Open Heart. (2022) 9:e002171. doi: 10.1136/openhrt-2022-002171, 36522127 PMC9756291

[40] Mehrabani S Bagherniya M Askari G Read MI Sahebkar A. The effect of fasting or calorie restriction on mitophagy induction: a literature review. J Cachexia Sarcopenia Muscle. (2020) 11:1447–58. doi: 10.1002/jcsm.12611, 32856431 PMC7749612

[41] Nakamura Y Walker BR Ikuta T. Systematic review and meta-analysis reveals acutely elevated plasma cortisol following fasting but not less severe calorie restriction. Stress. (2016) 19:151–7. doi: 10.3109/10253890.2015.1121984, 26586092

[42] Steinhauser ML Olenchock BA O'Keefe J Lun M Pierce KA Lee H . The circulating metabolome of human starvation. JCI Insight. (2018) 3:e121434. doi: 10.1172/jci.insight.121434, 30135314 PMC6141167

[43] Pilis K Godlewska U Pilis A Stec K Dolibog P Kruszewski M . Metabolic and hormonal effects of an 8 days water only fasting combined with exercise in middle aged men. Sci Rep. (2025) 15:22805. doi: 10.1038/s41598-025-05164-0, 40596030 PMC12219608

[44] Shkorfu W Fadel A Hamsho M Ranneh Y Shahbaz HM. Intermittent fasting and hormonal regulation: pathways to improved metabolic health. Food Sci Nutr. (2025) 13:e70586. doi: 10.1002/fsn3.70586, 40777199 PMC12330278

[45] Stapel B Fraccarollo D Westhoff-Bleck M Bauersachs J Lichtinghagen R Jahn K . Impact of fasting on stress systems and depressive symptoms in patients with major depressive disorder: a cross-sectional study. Sci Rep. (2022) 12:7642. doi: 10.1038/s41598-022-11639-1, 35538177 PMC9091273

[46] Tanner JM Kearns DT Kim BJ Sloan C Jia Z Yang T . Fasting-induced reductions in cardiovascular and metabolic variables occur sooner in obese versus lean mice. Exp Biol Med (Maywood). (2010) 235:1489–97. doi: 10.1258/ebm.2010.010171, 21127345 PMC3367312

[47] Srivastava N Kizhakkevalappil SP Gupta S Tyagi A Srivastava S. Effect of intermittent fasting on cardiovascular autonomic regulation in healthy adults. J Heart Valve Dis. (2025) 30:9–12.

[48] Wan R Weigand LA Bateman R Griffioen K Mendelowitz D Mattson MP. Evidence that BDNF regulates heart rate by a mechanism involving increased brainstem parasympathetic neuron excitability. J Neurochem. (2014) 129:573–80. doi: 10.1111/jnc.12656, 24475741 PMC4137462

[49] Lommatzsch M Zingler D Schuhbaeck K Schloetcke K Zingler C Schuff-Werner P . The impact of age, weight and gender on BDNF levels in human platelets and plasma. Neurobiol Aging. (2005) 26:115–23. doi: 10.1016/j.neurobiolaging.2004.03.002, 15585351

[50] Park S Zhang T Wu X Yi Qiu J. Ketone production by ketogenic diet and by intermittent fasting has different effects on the gut microbiota and disease progression in an Alzheimer's disease rat model. J Clin Biochem Nutr. (2020) 67:188–98. doi: 10.3164/jcbn.19-87, 33041517 PMC7533860

[51] Newman JC Verdin E. β-Hydroxybutyrate: a signaling metabolite. Annu Rev Nutr. (2017) 37:51–76. doi: 10.1146/annurev-nutr-071816-064916, 28826372 PMC6640868

[52] Gómora-García JC Montiel T Hüttenrauch M Salcido-Gómez A García-Velázquez L Ramiro-Cortés Y . Effect of the ketone body, D-β-Hydroxybutyrate, on Sirtuin2-mediated regulation of mitochondrial quality control and the autophagy-lysosomal pathway. Cells. (2023) 12:486. doi: 10.3390/cells12030486, 36766827 PMC9914182

[53] Hu E Du H Zhu X Wang L Shang S Wu X . Beta-hydroxybutyrate promotes the expression of BDNF in hippocampal neurons under adequate glucose supply. Neuroscience. (2018) 386:315–25. doi: 10.1016/j.neuroscience.2018.06.036, 29966721

[54] Lin J Ren Q Zhang F Gui J Xiang X Wan Q. D-β-Hydroxybutyrate dehydrogenase mitigates diabetes-induced atherosclerosis through the activation of Nrf2. Thromb Haemost. (2023) 123:1003–15. doi: 10.1055/s-0043-1770985 37399841

[55] Long YC Zierath JR. AMP-activated protein kinase signaling in metabolic regulation. J Clin Invest. (2006) 116:1776–83. doi: 10.1172/JCI29044, 16823475 PMC1483147

[56] Khalafi M Habibi Maleki A Mojtahedi S Ehsanifar M Rosenkranz SK Symonds ME . The effects of intermittent fasting on inflammatory markers in adults: a systematic review and pairwise and network Meta-analyses. Nutrients. (2025) 17:2388. doi: 10.3390/nu17152388, 40805975 PMC12348594

[57] Mulas A Cienfuegos S Ezpeleta M Lin S Pavlou V Varady KA. Effect of intermittent fasting on circulating inflammatory markers in obesity: a review of human trials. Front Nutr. (2023) 10:1146924. doi: 10.3389/fnut.2023.1146924, 37139450 PMC10149732

[58] de Wilhelmi Toledo F Grundler F Goutzourelas N Tekos F Vassi E Mesnage R . Influence of long-term fasting on blood redox status in humans. Antioxidants. (2020) 9:496. doi: 10.3390/antiox9060496, 32517172 PMC7346198

[59] Djurhuus CB Gravholt CH Nielsen S Mengel A Christiansen JS Schmitz OE . Effects of cortisol on lipolysis and regional interstitial glycerol levels in humans. Am J Physiol Endocrinol Metab. (2002) 283:E172–7. doi: 10.1152/ajpendo.00544.2001, 12067858

[60] Youm YH Nguyen KY Grant RW Goldberg EL Bodogai M Kim D . The ketone metabolite β-hydroxybutyrate blocks NLRP3 inflammasome-mediated inflammatory disease. Nat Med. (2015) 21:263–9. doi: 10.1038/nm.3804, 25686106 PMC4352123

[61] Yang Y Wang H Kouadir M Song H Shi F. Recent advances in the mechanisms of NLRP3 inflammasome activation and its inhibitors. Cell Death Dis. (2019) 10:128. doi: 10.1038/s41419-019-1413-8, 30755589 PMC6372664

[62] Ahn JH Shin BN Song M Kim H Park JH Lee TK . Intermittent fasting increases the expressions of SODs and catalase in granule and polymorphic cells and enhances neuroblast dendrite complexity and maturation in the adult gerbil dentate gyrus. Mol Med Rep. (2019) 19:1721–7. doi: 10.3892/mmr.2019.9822, 30628688 PMC6390044

[63] Tavakoli A Akhgarjand C Ansar H Houjaghani H Khormani A Djafarian K . The effects of intermittent fasting on antioxidant and inflammatory markers and liver enzymes in postmenopausal, overweight and obese women with rheumatoid arthritis: a randomized controlled trial. Sci Rep. (2025) 15:2357. doi: 10.1038/s41598-025-86734-0, 39825120 PMC11742681

[64] Hirabayashi T Nakanishi R Tanaka M Nisa BU Maeshige N Kondo H . Reduced metabolic capacity in fast and slow skeletal muscle via oxidative stress and the energy-sensing of AMPK/SIRT1 in malnutrition. Phys Rep. (2021) 9:e14763. doi: 10.14814/phy2.14763, 33650806 PMC7923585

[65] Xu Z Han X Ou D Liu T Li Z Jiang G . Targeting PI3K/AKT/mTOR-mediated autophagy for tumor therapy. Appl Microbiol Biotechnol. (2020) 104:575–87. doi: 10.1007/s00253-019-10257-8, 31832711

[66] Zenz G Jačan A Reichmann F Farzi A Holzer P. Intermittent fasting exacerbates the acute immune and behavioral sickness response to the viral mimic poly(I:C) in mice. Front Neurosci. (2019) 13:359. doi: 10.3389/fnins.2019.00359, 31057355 PMC6478699

[67] Braz AF Figueira de Oliveira ML Costa DHSD Torres-Leal FL Guedes RCA. Treadmill exercise reverses the adverse effects of intermittent fasting on behavior and cortical spreading depression in young rats. Brain Sci. (2023) 13:1726. doi: 10.3390/brainsci13121726, 38137174 PMC10742290

[68] Hu H Li F Cheng S Qu T Shen F Cheng J . Alternate-day fasting ameliorated anxiety-like behavior in high-fat diet-induced obese mice. J Nutr Biochem. (2024) 124:109526. doi: 10.1016/j.jnutbio.2023.109526, 37931668

[69] Soares NL Cavalcante HC Ferreira SL do Nascimento FBL Costa IKC Rolim TBB . Different intermittent fasting regimens decrease anxious and depressive-like behaviour, but show damage to brain tissues in healthy Wistar rats. Nutr Neurosci. (2025) 28:1585–602. doi: 10.1080/1028415X.2025.2533967, 40684295

[70] Nugraha B Ghashang SK Hamdan I Gutenbrunner C. Effect of Ramadan fasting on fatigue, mood, sleepiness, and health-related quality of life of healthy young men in summer time in Germany: a prospective controlled study. Appetite. (2017) 111:38–45. doi: 10.1016/j.appet.2016.12.030, 28027907

[71] Nugraha B Riat A Ghashang SK Eljurnazi L Gutenbrunner C. A prospective clinical trial of prolonged fasting in healthy young males and females-effect on fatigue, sleepiness, mood and body composition. Nutrients. (2020) 12:2281. doi: 10.3390/nu12082281, 32751487 PMC7469051

[72] Riat A Suwandi A Ghashang SK Buettner M Eljurnazi L Grassl GA . Ramadan fasting in Germany (17-18 h/day): effect on cortisol and brain-derived neurotrophic factor in association with mood and body composition parameters. Front Nutr. (2021) 8:697920. doi: 10.3389/fnut.2021.697920, 34458302 PMC8387581

[73] Marciniak M Sato M Rutkowski R Zawada A Juchacz A Mahadea D . Effect of the one-day fasting on cortisol and DHEA daily rhythm regarding sex, chronotype, and age among obese adults. Front Nutr. (2023) 10:1078508. doi: 10.3389/fnut.2023.1078508, 36814510 PMC9940638

[74] Vance ML Thorner MO. Fasting alters pulsatile and rhythmic cortisol release in normal man. J Clin Endocrinol Metab. (1989) 68:1013–8. 2723024 10.1210/jcem-68-6-1013

[75] Bains G Lohman E Moh M Daher N Berk L. Four weeks of 16:8 intermittent fasting on stress, sleep, quality of life, and body composition in healthy adults: a pilot study on wellness. J Wellness. (2021) 3. doi: 10.18297/jwellness/vol3/iss2/10

[76] Clavero-Jimeno A Dote-Montero M Migueles JH Camacho-Cardenosa A Medrano M Alfaro-Magallanes VM . Time-restricted eating and sleep, mood, and quality of life in adults with overweight or obesity: a secondary analysis of a randomized clinical trial. JAMA Netw Open. (2025) 8:e2517268. doi: 10.1001/jamanetworkopen.2025.17268, 40560588 PMC12199060

[77] Huo L Li Y Fu Y Yang Z Jia L Li C . Effects of intermittent fasting on anxiety and the functional connectivity of the amygdala in healthy adults. Alpha Psychiatry. (2025) 26:44384. doi: 10.31083/AP44384, 40630882 PMC12231414

[78] Abdelrahim DN Rachida R Krami AM Nadia A Faris ME. Sex as a biological determinant in anthropometric, biochemical, and dietary changes during Ramadan intermittent fasting in healthy people: a systematic review. Diabetes Metab Syndr. (2023) 17:102762. doi: 10.1016/j.dsx.2023.102762 37141819

[79] Alum E Obeagu E Ugwu PC Alum B Echegu A Ukaidi C. Differential impacts of intermittent fasting on men and women. Elite J Health Sci. (2024) 2:37–44.

[80] Soeters MR Sauerwein HP Groener JE Aerts JM Ackermans MT Glatz JF . Gender-related differences in the metabolic response to fasting. J Clin Endocrinol Metab. (2007) 92:3646–52. doi: 10.1210/jc.2007-0552, 17566089

[81] Sudasinghe KH White ZJ Hall SE. Sexual dimorphism in response to intermittent fasting and its impact on the brain. Sci Rep. (2025) 15:24846. doi: 10.1038/s41598-025-09692-7, 40640397 PMC12246044

[82] Yanko R. Age-related differences in the effect of intermittent fasting on the morphofunctional parameters of the rat’s pancreas. TJOVNKKNUSB. (2024) 43:138–44. doi: 10.26565/2075-5457-2024-43-12

[83] Sadowska J Dudzińska W Dziaduch I. The effect of alternating high-sucrose and sucrose free-diets, and intermittent one-day fasting on the estrous cycle and sex hormones in female rats. Nutrients. (2022) 14:4350. doi: 10.3390/nu14204350, 36297033 PMC9611605

[84] Bergendahl M Iranmanesh A Mulligan T Veldhuis JD. Impact of age on cortisol secretory dynamics basally and as driven by nutrient-withdrawal stress. J Clin Endocrinol Metab. (2000) 85:2203–14. 10852453 10.1210/jcem.85.6.6628

[85] Merikanto I Lahti T Puolijoki H Vanhala M Peltonen M Laatikainen T . Associations of chronotype and sleep with cardiovascular diseases and type 2 diabetes. Chronobiol Int. (2013) 30:470–7. doi: 10.3109/07420528.2012.741171, 23281716

[86] Almoosawi S Vingeliene S Gachon F Voortman T Palla L Johnston JD . Chronotype: implications for epidemiologic studies on Chrono-nutrition and Cardiometabolic health. Adv Nutr. (2019) 10:30–42. doi: 10.1093/advances/nmy070, 30500869 PMC6370261

[87] Romanenko M Schuster J Piven L Synieok L Dubiley T Bogomaz L . Association of diet, lifestyle, and chronotype with metabolic health in Ukrainian adults: a cross-sectional study. Sci Rep. (2024) 14:5143. doi: 10.1038/s41598-024-55715-0, 38429516 PMC10907368

[88] Lu L Chen X Liou S Weng X. The effect of intermittent fasting on insulin resistance, lipid profile, and inflammation on metabolic syndrome: a GRADE assessed systematic review and meta-analysis. J Health Popul Nutr. (2025) 44:293. doi: 10.1186/s41043-025-01039-2, 40826125 PMC12363089

[89] Zhang S Sun B Sun L Zou S Chen Q. Effect of intermittent fasting on obesity and metabolic indices in patients with metabolic syndrome: a systematic review and meta analysis. BMC Endocr Disord. (2025) 25:130. doi: 10.1186/s12902-025-01952-x, 40369509 PMC12076832

[90] Entezari M Hashemi D Taheriazam A Zabolian A Mohammadi S Fakhri F . AMPK signaling in diabetes mellitus, insulin resistance and diabetic complications: a pre-clinical and clinical investigation. Biomed Pharmacother. (2022) 146:112563. doi: 10.1016/j.biopha.2021.112563, 35062059

[91] Cramer H Hohmann C Lauche R Choi KA Schneider N Steckhan N . Effects of fasting and lifestyle modification in patients with metabolic syndrome: a randomized controlled trial. J Clin Med. (2022) 11:4751. doi: 10.3390/jcm11164751 36012990 PMC9410059

[92] Fu W Yang K Tang R Wang J Liu Y Liu G . Effects of time-restricted eating on glycemic control in type 2 diabetes: a 12-week quasi-experimental single-arm study with 1-year follow-up. Clin Nutr. (2025) 52:263–74. doi: 10.1016/j.clnu.2025.08.002, 40812266

[93] Knufinke M Lebbing M Mesnage R. Case report: sustained weight loss and glycemic control from repeated long-term fasting in type 2 diabetes. Front Clin Diabetes Healthc. (2025) 6:1572245. doi: 10.3389/fcdhc.2025.1572245, 40851781 PMC12367449

[94] Keenan S Cooke MB Belski R. The effects of intermittent fasting combined with resistance training on lean body mass: a systematic review of human studies. Nutrients. (2020) 12:2349. doi: 10.3390/nu12082349, 32781538 PMC7468742

[95] Gul F Herrema H Ameer A Davids M Nasir A Gerasimidis K . Dietary composition and fasting regimens differentially impact the gut microbiome and short-chain fatty acid profile in a Pakistani cohort. Front Syst Biol. (2025) 5:1622753. doi: 10.3389/fsysb.2025.1622753, 41179493 PMC12575385

[96] Angoorani P Ejtahed H-S Hasani-Ranjbar S Siadat SD Soroush AR Larijani B. Gut microbiota modulation as a possible mediating mechanism for fasting-induced alleviation of metabolic complications: a systematic review. Nutr Metabol. (2021) 18:105–17. doi: 10.1186/s12986-021-00635-3, 34906176 PMC8670288

[97] Pramono A Ardiaria M Limijadi EKS Noer ER Lestari ES Siswanto FM. Intermittent fasting modulates human gut microbiota diversity in a phenotype-dependent manner: a systematic review. Biosci Microbiota Food Health. (2024) 43:170–82. doi: 10.12938/bmfh.2023-111, 38966051 PMC11220331

[98] Abdel-Rahman M Hussein AA Ahmed-Farid OA Sawi AA Abdel Moneim AE. Intermittent fasting alerts neurotransmitters and oxidant/antioxidant status in the brain of rats. Metab Brain Dis. (2024) 39:1291–305. doi: 10.1007/s11011-024-01415-7, 39292431 PMC11513736

[99] Wan Z Li S Fang S. The effect of negative physical self on social anxiety in college students: the bidirectional chain mediation roles of fear of negative evaluation and regulatory emotional self-efficacy. Psychol Res Behav Manag. (2024) 17:2055–66. doi: 10.2147/PRBM.S457405 38800523 PMC11122180

[100] Calabrese EJ Mattson MP. How does hormesis impact biology, toxicology, and medicine? npj Aging Mech Dis. (2017) 3:13. doi: 10.1038/s41514-017-0013-z 28944077 PMC5601424

